# Impact of Body Mass Index on Survival of Metastatic Renal Cancer

**DOI:** 10.15586/jkcvhl.v8i2.169

**Published:** 2021-07-31

**Authors:** Jose Javier Salgado Plonski, Sergio Fernández-Pello, Laura Rúger Jiménez, Iván González Rodríguez, Laura Alonso Calvar, Luis Rodríguez Villamil

**Affiliations:** Department of Urology, Hospital Universitario de Cabueñes, Gijón, Spain

**Keywords:** body mass index, carcinoma, metastatic, renal, survival

## Abstract

Obesity has been established as a risk factor for renal cell carcinoma (RCC). Recently, studies have described obesity as a probable protecting factor in the metastatic stage of RCC. In this study, we assessed the relationship between body mass index (BMI) and overall survival in patients under systemic therapy.

The correlation between BMI and overall median survival was studied in 76 patients diagnosed with metastatic RCC under systemic therapy. The groups were divided into overweight and obesity (BMI > 25 kg/m^2^) and underweight or normal (BMI < 25 kg/m^2^). Statistical analysis was performed using the Cox regression model adjusted by gender.

A total of 76 patients were studied: 16 women (21%) and 60 men (79%). The median BMI was 27.96 kg/m^2^; 24 patients (31.6%) had low BMI and 52 (68.4%) had high BMI. Median overall survival in the group with BMI > 25 kg/m^2^ was 17 months (95% confidence interval [CI]: 13–34 months), while in the group with BMI ≤ 25 kg/m^2^, it was 14 months (95% CI: 8–20 months). When adjusted by gender, the group with BMI > 25 kg/m^2^ presented a hazards ratio of 0.54 (95% CI: 0.30–0.96), P = 0.044 (Log Rank).

A high BMI significantly acts as a protecting factor. We observed an increased overall survival of overweight and obese patients within the context of metastatic RCC under systemic treatment. These data confirm the findings published in other studies that suggest the role of lipid metabolism in this type of tumors.

## Introduction

Obesity is a major worldwide health condition linked to angiogenic-related diseases such as diabetes, cardiovascular events, and cancer (1). Although there is a clear trend between diabetes, heart diseases, and obesity, the relationship between obesity and cancer is confusing, being a risk factor in some aspects and a protective factor in others (1, 2).

Some angiogenic growth factors and hormones are produced by fatty tissues (3), including vascular and endothelial growth factor (VEGF), tumoral necrosis factor (TNF), and leptin (4). These factors derived from fatty cells have a crucial role in tissue and organ regulation due to angiogenic checkpoint control; however, the overexpression of these factors could not be sufficient to boost it because the feedback control of many other activators and inhibitors (5).

Currently, the mechanisms that connect overweight and obesity to different cancer subtypes have been inconclusive. It is known that overweight and obesity can cause potential changes in hormonal metabolism (insulin, Insulin-like Growth Factor-1 [IGF-1], and sexual steroids), and proteins produced by fatty tissues (adipokines) are also involved in immune regulation (leptin), inflammatory response (TNFα, interleukin-6 [IL-6], and amyloid A), stromal response, and angiogenesis (VEGF-1) as well as extracellular matrix components (collagen type 6 [Col VI]) (6).

Although it is reported that overweight and obesity are risk factors in the development of Renal Cell Carcinoma (RCC), different studies have found a “paradoxical” relationship between high body mass index (BMI) and improved overall survival (OS), and between cancer-specific survival (CSS) and relapse-free survival (RFS) compared to lower BMI patients (7).

Although a clear cause has not been found to explain this “paradoxical” relationship, there is a hypothesis that proposes the power of lipid metabolism over tumor cells (2). Several studies have stated that there is an inverse correlation between BMI and adiponectin serum levels in RCC. In this sense adiponectin is a polypeptide elaborated by the fatty tissue and the higher levels could be explained by aggressive features and less survival in the context of patients (8). All these studies have concluded that adiponectin favors proliferation and checkpoint proteins, and inhibits apoptosis and angiogenesis (8, 9).

In the largest series which studied the impact of BMI on metastatic RCC patients treated with systemic therapy, according to the International Metastatic Renal Cell Cancer Database Consortium (IMDC) data, the overall survival significantly improved in the overweight patients group (BMI ≥ 25 kg/m^2^) compared to normal-weight or low-weight group (BMI < 25 kg/m^2^). The study concluded that overweight and obesity behave as independent prognostic factors, and this prognostic condition was kept across all IMDC prognostic subgroups (10–12).

Our objective was to explain the relationship that exists between BMI and overall survival in our metastatic RCC patients treated with systemic therapy based on tyrosine kinase inhibitors (TKI) and rapamycin inhibitors (mTOR). The primary endpoint is the measure of overall survival based on BMI levels. The secondary endpoint is the descriptive analysis of clinic and pathologic features of these patients.

## Material and Methods

This is a single center retrospective cohort study with survival analysis performed in a regional secondary level hospital, where physicians of the Urology team studied metastatic RCC Caucasian patients treated with systemic therapy between 2006 and 2017. The median follow-up time was 58 months (14 months–118 months). Patients received treatment with sunitinib, pazopanib, temsirolimus, everolimus, sorafenib, axitinib, nivolumab, or bevacizumab plus Interferon (IFN) alfa. According to the World Health Organization BMI classification, a cutoff value of 25 kg/m^2^ was used to stratify patients between under or normal weight (BMI < 25 kg/m^2^) (13) and overweight or obesity (BMI ≥ 25 kg/m^2^) in different grades in order to make subgroups to analyze the relationship of BMI with the median overall survival. According to the Memorial Sloan-Kettering Cancer Center (MSKCC) classification, the studied variables were as follows: age, gender, previous nephrectomy, histology subgroup, and risk stratification (14). Both groups did not show any significant differences except the BMI.

The statistical analysis was performed by using the Cox regression model, adjusted by gender. The Kaplan–Meier model was used to express survival curves with forest plot diagram to study obesity as a prognostic factor. Median overall survival was considered as the time from diagnosis of metastatic RCC until the death of the patient in the context of tumor progression. Confidence Interval (CI) at 95% (95% CI) and P < 0.05 were considered statistically significant. SPSS version 25.0 (IBM Corp., released 2017, version 25.0, Armonk, NY) was used for statistical analysis.

The study was deemed exempt from approval of research ethics committee (Servicio de Salud del Principado de Asturias decision tool). As only retrospectively collected clinical data was used for analysis, informed consent was not sought from patients. The research was conducted in a responsible and ethical manner according to accepted standards and the study was performed in line with the principles of the Declaration of Helsinki.

## Results

In all, 76 metastatic RCC patients treated with systemic treatment with target therapies were included. Demographic, clinical, and pathologic features are described in Table 1.

**Table 1:** Features analyzed according to BMI groups.

Clinicopathologic and demographic features	Total (n = 76)	No. of Patients (%)	
		BMI* ≤ 24.9 kg/m^2^ (normal or under weight) (n = 23)	BMI* > 25 kg/m^2^ overweight, obesity (n = 53)
*Age*			
<60 years	18 (23.68)	5 (21.73)	14 (26.41)
>60 years	58 (76.32)	18 (78.26)	39 (73.58)
*Gender*			
Male	60 (78.94)	17 (73.91)	43 (81.13)
Female	16 (21.05)	6 (26.08)	10 (18.86)
*Nephrectomy*			
Yes	59 (77.63)	19 (82.60)	41 (77.35)
No	17 (22.36)	4 (17.39)	12 (22.64)
*Pathologic features*			
Clear cell carcinoma	70 (92.10)	19 (82.60)	51 (96.22)
Papillary	5 (6.57)	3 (13.04)	2 (3.77)
Chromophobe	1 (1.31)	1 (4.34)	0 (0)
MSKCC **classification (15)			
Favorable	22 (28.94)	5 (21.73)	17 (32.07)
Intermediate	49 (64.47)	16 (69.56)	33 (62.26)
Poor	5 (6.57)	2 (8.69)	3 (5.66)
IMDC*** classification (13)			
Favorable	27 (35.5)	7 (25.92)	20 (74.07)
Intermediate	37 (48.68)	12 (32.43)	25 (67.56)
Poor	12 (15.78)	7 (58.33)	5 (41.66)

* Body Mass Index, **Memorial Sloan Kettering Cancer Center, ***International Metastasic Renal Cell Carcinoma Database Consortium.

According to the Response Evaluation Criteria in Solid Tumors (RECIST), the objective response rate was 36% in the first line and 14% for the second line of treatment.

The Kaplan–Meier model analysis revealed a median overall survival of 17 months in the group with BMI > 25 kg/m^2^ (95% CI: 13–34), while in the group with BMI ≤ 25 kg/m^2^, it was 14 months (95% CI: 8–20). Adjusting the groups by gender, the differences in overall survival between them showed statistically significant differences (P = 0.044; Table 2).

**Table 2:** Median overall survival according to BMI groups.

Features	Median overall survival (months)	95% CI (months)	
BMI* ≤ 24.9 kg/m^**2**^ (normal or under weight)	14	8–20	
BMI* > 25 kg/m^**2**^ (overweight, obesity)	17	13–34	
P-value			0.044

* Body Mass Index

**Figure 1:**
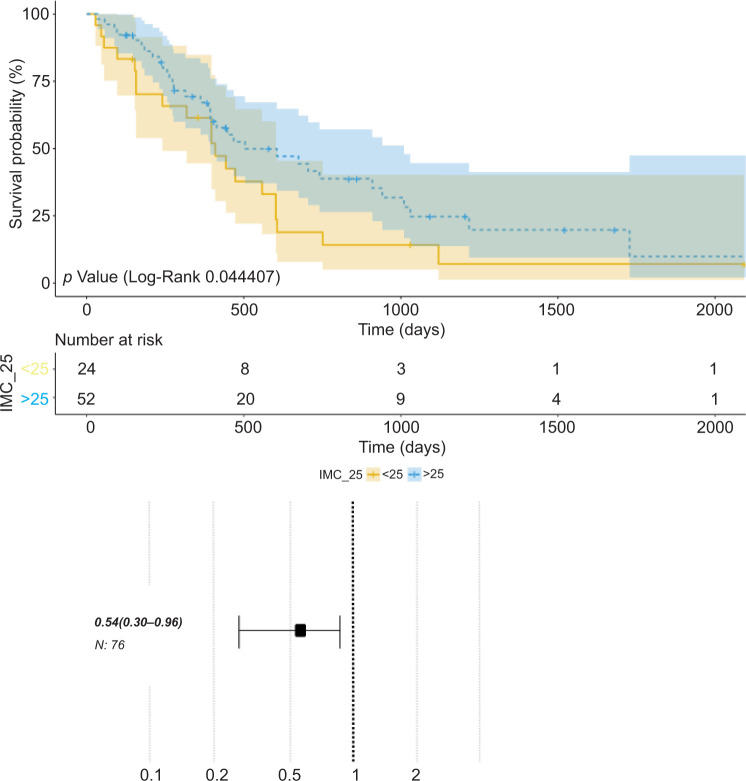
The Kaplan–Meier analysis. Hazards ratio of 0.54 (CI 95%: 0.30–0.96).

The risk analysis of the impact of obesity on the overall survival of metastatic RCC patients showed a risk ratio of 0.54 (95% CI: 0.30–0.96), considering overweight and obesity as protective factors in this context (Figure 1).

## Discussion

Obesity has been described as a clear and significant risk factor for the incidence of local RCC being present in 30–40% of all cases (2), but in the metastatic scenario this factor could behave in a different way. There are multiple studies that have shown that a high BMI is related to an increase in the survival of patients with metastatic RCC. They have concluded that overweight and obesity behave as independent prognostic factors (2, 10–12). The largest systematic review and meta-analysis that has investigated the association between BMI and oncologic outcomes supported the evidence that an “obesity paradox” is demonstrated in cancer-specific survival, overall survival, and relapse-free survival (2).

Several hypotheses have been described but the exact mechanisms of how obesity acts to protect patients with metastatic RCC are unknown. In the lipogenesis pathway, fatty acid synthetase (FASN) has been studied as an involved key enzyme. FASN has emerged as a metabolic oncogene with an important role in the growth and survival of tumors (10). A high BMI has been associated with a low expression of *FASN* gene and the expression of FASN (using median as a cut-off point) has been inversely associated with overall survival in patients receiving first- and second-line of treatment for metastatic RCC after adjusting of prognostic characteristics (11, 12). A fundamental role of perinephric fat in modulating tumor response acting as a reservoir of activated cells that can be mobilised through the administration of systemic therapies has been described. Microenvironment in tumors with higher angiogenesis scores makes these tumors more susceptible to tyrosine kinase inhibitors. This could contribute to apparent survival advantage in obese patients with clear cell RCC compared to patients having normal weight (14, 16, 17).

Another hypothesis is that obese patients may have a higher prevalence of comorbidities that could lead to perform image studies, which can lead to a higher rate of incidental and early detection of small renal tumors. The treatment of small renal tumors could achieve better control of the disease, including the metastatic stage.

We studied the relationship between BMI and overall survival in patients with metastatic RCC having systemic therapy with different lines of treatment. Results of our study demonstrated that there was a statistically significant increase in overall survival in overweight and obese patients with metastatic RCC compared to those having normal or low weight according to BMI groups. These data confirm the findings proposed in other studies that suggest the role of lipid metabolism in this type of tumors (11, 12).

In a descriptive analysis of demographic, clinical, and pathologic characteristics of patients, our data are related to previously published epidemiologic studies (1).

## Limitations

We fairly admit some limitations to this study. First limitation is the limited number of patients. The retrospective design could include potential and inherent bias because of remarked nature of investigation. In addition, lack of adjustment within the study groups regarding pathologic and demographics could influence the overall conclusions. Overlap between confidence intervals could limit statistical significance in spite of P-value levels.

This study could lead to the future studies to evaluate the prognostic impact of BMI in patients with metastatic RCC undergoing systemic therapy in a larger cohort. The future study with adequate design, including metabolic biomarkers such as leptin or adiponectin, must investigate the impact of adipokines on BMI to understand the biologic mechanisms responsible for the increased survival in these patients. The use of imaging tests, such as computed tomography, could benefit in the precision of correct analysis of body composition compared to BMI.

## Conclusions

In conclusion, a high BMI behaves as a protective factor with increased overall survival in patients with metastatic RCC under systemic treatment.
